# Highlight—Lessons on Parasitism from the Curious Dicyemida

**DOI:** 10.1093/gbe/evz160

**Published:** 2019-08-21

**Authors:** Casey McGrath

The incredible diversity of life forms on the planet led Charles Darwin to note: “From so simple a beginning endless forms most beautiful and most wonderful have been, and are being, evolved.” In order to gain a true understanding of the power and limitations of evolution to produce such “endless forms,” it is important to study a variety of organisms from across the tree of life. Luckily, next-generation sequencing technologies have enabled in-depth genomic analysis of virtually any organism, expanding the scope of powerful evolutionary analyses beyond model organisms. For example, take the decidedly unusual animal, the Dicyemida.

**Fig. 1.**
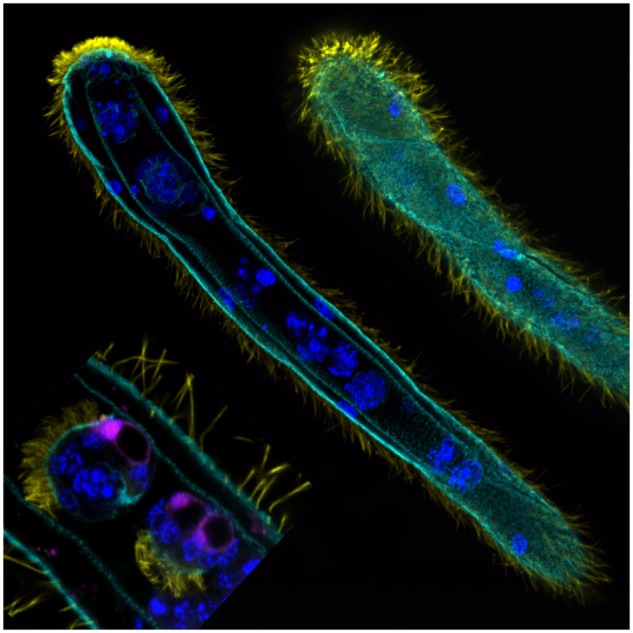
—Confocal image showing the surface and cross-section of a rhombogen adult dicyemid and infusoriform dicyemid embryos developing inside a rhombogen. Image by Tsai-Ming Lu.

Dicyemida are microscopic organisms consisting of only ∼30 cells each (Fig. 1). They are parasites that live inside the renal sacs of cephalopods like the squid and octopus, deriving nutrients from the host’s urine. Despite a relatively complex lifecycle, dicyemids lack differentiated tissues such as a digestive tract and circulatory system and thus represent one of the most extreme cases of body plan reduction among animals. Because of their small size and simplified tissues, they were once thought to represent a link between single-celled and multicellular organisms. However, their true phylogenetic position was long considered controversial and was only resolved in 2017 when PhD student Tsai-Ming Lu at Okinawa Institute of Science and Technology Graduate University, along with Drs Miyuki Kanda, Noriyuki Satoh, and Hidetaka Furuya, used transcriptomic data to definitively place Dicyemida within the group Spiralia, along with segmented worms, flatworms, and mollusks (Lu et al. 2017).

Now, the same group of researchers has extended their analysis of this lineage by sequencing the complete genome of *Dicyema japonicum.* The results are published in this issue of *Genome Biology and Evolution* in the article, “Dicyemid mesozoans: a unique parasitic lifestyle and a reduced genome” (Lu et al. 2019), providing further insight into this odd organism and revealing convergent patterns that are shared among parasites in general.

Parasitism has been observed in 15 of the 35 animal phyla and is thought to have evolved independently over 200 times. While each parasitism event reflects a specific interaction between a host and parasite, parasites often possess certain features—such as a simplified body plan and complex lifecycle—that reflect the selective pressures commonly experienced by organisms that “make a living” off of other organisms. In order to understand the evolution of parasitism, the authors sought to explore the genomic innovations that make this lifestyle possible. According to the authors, “Decoding the dicyemid genome not only improves our understanding of the biology of dicyemids, such as their complicated lifecycle, but also provides us a rich resource for comparative studies to [better understand] the evolution of spiralians from a genomic perspective.”

With these aims in mind, sequencing of the genome of *D. japonicum* revealed that, like other parasites, it has a reduced genome, with only ∼5,000 genes. This includes only four Hox genes, highly conserved genes crucial for delineating the animal body plan. In particular, the authors note, “Compared with reduced sets of genes in other spiralian parasites, dicyemids possess fewer genes and an extraordinarily shortened intron size” (∼38 bp). Reduced genomes tend to be widespread among parasites, allowing them to limit energy consumption by decreasing the amount of genetic material that must be maintained.

To determine how evolution drove the loss of genetic material, the researchers investigated which genes were retained or lost on a genome-wide scale. Rather than eliminating entire metabolic pathways, it appears that individual *Dicyema* genes were lost from a variety of pathways, resulting in streamlined and simplified pathways. With regard to the genes that were retained, the authors note that “the retention of some genes may indicate that their fundamental functions are shared by multiple pathways.” However, this raises the questions of which pathways remain functional despite being incomplete and how dicyemids overcome the physiological gaps caused by lost genes.

Answering questions about the fine-scale changes that have occurred in various metabolic pathways will require further studies of dicyemid physiology. As pointed out by the authors, however, such studies are complicated by the unique biology of dicyemids: “In addition to operational difficulties caused by the tiny size of dicyemids, currently, methods to manipulate gene expression have not been established in dicyemids, so it is still difficult to execute functional tests.” Despite the potential challenges, the genome sequence of this unusual parasite represents a resource that will be useful for future analyses, providing molecular information that could be used to reveal the ways in which this organism is similar to other spiralians and animal parasites, as well as the ways in which it is wholly unique.
